# Microhabitat use, daily activity pattern, and diet of *Liolaemus etheridgei* Laurent, 1998 (Reptilia: Liolaemidae) in the Andean *Polylepis* forests of Arequipa, Peru

**DOI:** 10.1002/ece3.9363

**Published:** 2022-10-01

**Authors:** Irbin B. Llanqui, Bryn Edwards, Evaristo López Tejeda

**Affiliations:** ^1^ Escuela de Ciencias Biológicas Universidad Nacional Mayor de San Marcos Lima Peru; ^2^ Lancaster Environment Centre Lancaster University Lancaster UK; ^3^ Escuela de Biología Universidad Nacional de San Agustín de Arequipa Arequipa Peru

**Keywords:** bimodal activity pattern, herbivory, *Liolaemus montanus* group, omnivore, resource selection

## Abstract

This study describes the microhabitat use, daily activity pattern, and diet of *Liolaemus etheridgei* Laurent, 1998 in the El Simbral and Tuctumpaya *Polylepis* forests in Arequipa, Peru. El Simbral is a fragmented forest, whereas Tuctumpaya is unfragmented. Our results reveal that *L. etheridgei* shows no positive selection for any of the microhabitats we identified in *Polylepis* forests; on the contrary, it selects negatively against *Polylepis* trees and nonthorny bushes. The daily activity patterns indicate a bimodal pattern with peaks at 9:00–10:59 and 13:00–13:59 h. The diet of *L. etheridgei* consists mainly of plant material, and the most important animal prey category is Lygaeidae: Hemiptera, which is selected for positively. In particular, microhabitat selection varied for nonthorny bushes, which were selected negatively in the Tuctumpaya population but neither positively nor negatively in the El Simbral population. According to the proportions of plant material found, the *L. etheridgei* from El Simbral were found to be omnivorous, whereas the Tuctumpaya population was herbivorous. However, the percentage of plant material consumed in the El Simbral population was close to the critical value for herbivory–omnivory. We conclude that the three ecological aspects of *L. etheridgei* studied here are virtually identical in El Simbral and Tuctumpaya; therefore, this species is not affected significantly by the current fragmentation of forest.

## Graphical abstract

Our study provides information on microhabitat use, daily activity pattern, and diet of *Liolaemus etheridgei*.

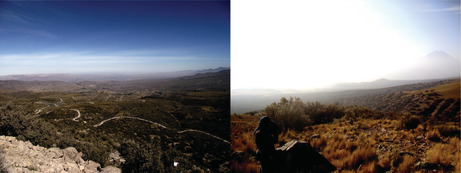

## 1 INTRODUCTION

The *Liolaemus* genus consists of a large group of lizard species distributed from the central Andes of Peru to Tierra del Fuego in Southern Chile and Argentina, within an elevational range of 0–5000 m.a.s.l. (Abdala et al., 2019; Lobo et al., 2010). Currently, there are 37 *Liolaemus* species recorded in Peru (Uetz & Hošek, 2022). It is also thought that *Liolameus* species richness in Peru is still underestimated, and it is likely that more species could be described in the future (Gutiérrez et al., 2018). However, there is still a lack of ecological studies for most of these species, which limits our knowledge of their population status and management requirements. Some insights into the ecology of *Liolaemus* species in Peru come from short natural history notes in taxonomical studies (but see Llanqui, 2020; Olivera Jara & Aguilar, 2020). For example, Chaparro et al. (2020) report an observation of *L. qalaywa* eating the amphibian *Pleurodema marmoratum* and some larval insects; Huamani‐Valderrama et al. (2020) report the consumption of coleopteran larvae and lepidopteran adults by *L. anqapuka*. Gutiérrez et al. (2018) report the consumption of Carabidae: Coleoptera, Aranea, and larvae by *L. evearistoi*. However, thorough ecological information is limited, especially when compared with studies on Argentinian or Chilean species. This is of particular concern for multiple *Liolaemus* species in Peru, such as *L. anqapuka, L. annectens* sensu lato, *L. insolitus*, *L. polystictus, L. qalaywa* and in general, the *L. montanus* species group, which are threatened permanently by agriculture, mining activities, urban expansion, habitat fragmentation, and climate change (Aguilar et al., 2017; Aguilar‐Puntriano et al., 2019; Chaparro et al., 2020; Huamani‐Valderrama et al., 2020; Olivera Jara & Aguilar, 2020). Thus, a better understanding of the ecology of these lizards is necessary in order to propose efficient conservation actions.

*Liolaemus etheridgei* is distributed in the Arequipa and Moquegua Regions in southern Peru (Laurent, 1998; Llanqui, 2020), occupying the scrubland, shrubland, and *Polylepis* woodland habitats found there. The *Polylepis* woodlands are present throughout the distribution of *L. etheridgei*, comprising small areas of relictual woodland. However, the role of these woodlands in sustaining *L. etheridgei* populations is unclear (see Lloyd & Marsden, 2008). Of the 19 *Polylepis* species recognized in Peru (Mendoza & Cano, 2011), 13 (68%) have been included in a threatened category nationally (Decreto Supremo 043‐2006‐AG. Available from https://www.serfor.gob.pe/portal/wp‐content/uploads/2016/03/D.S.‐N‐043‐2006‐AG‐Aprueban‐Categorizacin‐de‐Especies‐Amenazadas‐de‐Flora‐Silvestre.pdf [Accessed 24 April 2021]), urging further investigation into their decline. This study focused on two *Polylepis rugulosa* Bitter, 1911 (local name “Queñua”) relicts with differring levels of fragmentation: El Simbral and Tuctumpaya. El Simbral is a forest fragmented by the Moquegua‐Arequipa Road and with smaller trees, while Tuctumpaya remains unfragmented and with taller trees. In this study, we aim to describe three aspects of the ecology of *L. etheridgei* in *Polylepis* forests: microhabitat use, daily activity pattern, and diet. We will also compare the differences in these aspects between the populations inhabiting fragmented and unfragmented forests. Differences in these aspects could indicate changes due to habitat deterioration. We also focus on these aspects as their divergence can lead to the ecological diversification of *Liolaemus* species (Jaksić et al., 1980). For microhabitat use, we expect *L. etheridgei* populations to select *Polylepis* trees positively as they have commonly been thought to be an essential resource for these lizards (Gutiérrez et al., 2010). For the differences in daily activity patterns, we expect *L. etheridgei* to be active for longer in El Simbral due to a lower tree cover enabling higher insolation, thus maintaining adequate temperatures in the substrate for lizards to be active for longer. Finally, we expect the diet to be more diverse in the Tuctumpaya population due to a diverse community of prey species in the unfragmented forest.

## 2 MATERIAL AND METHODS

### 2.1 Study area

**FIGURE 1:**
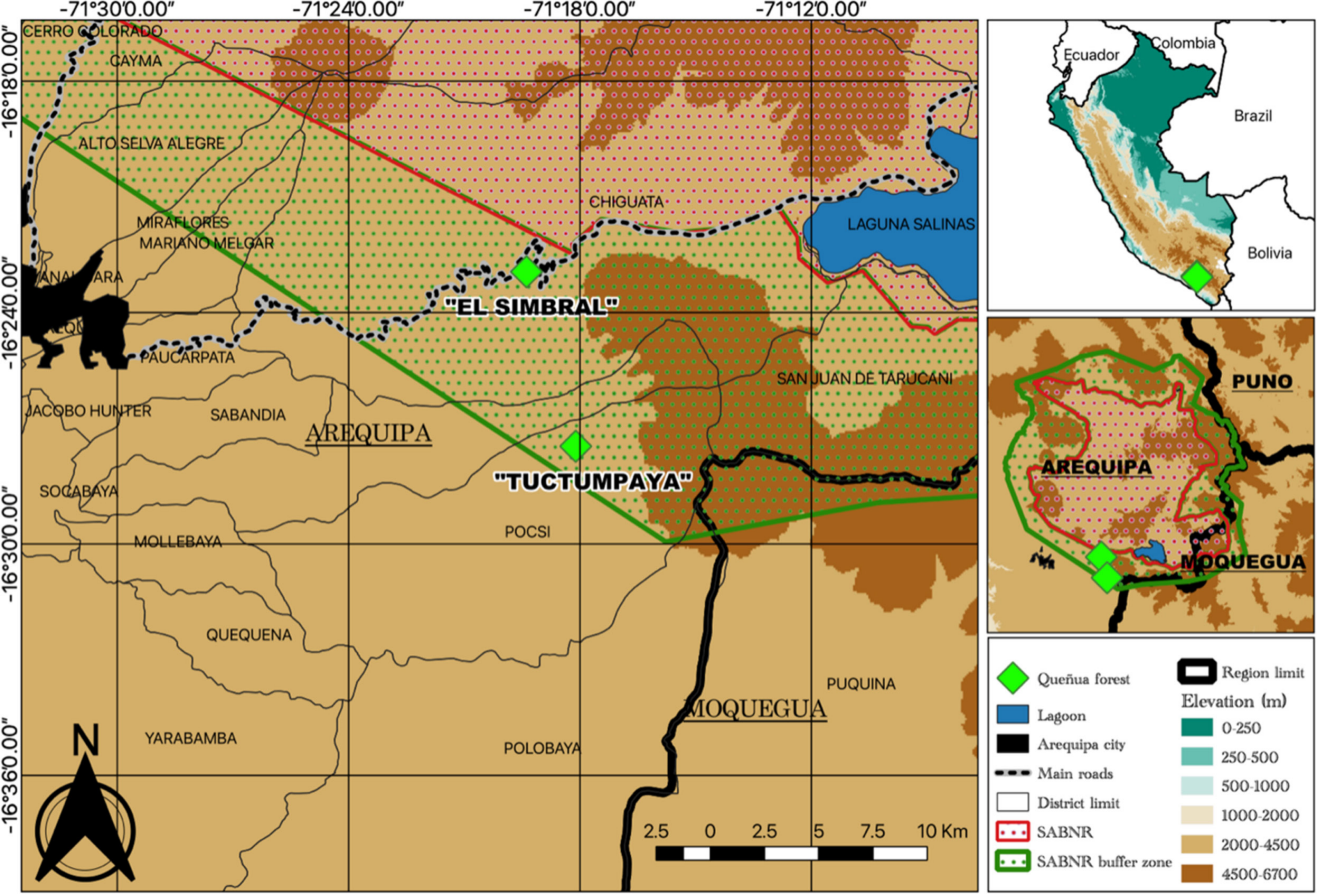
Location of the *Polylepis* forests of “El Simbral” and “Tuctumpaya” in the buffer zone of the Salinas y Aguada Blanca National Reserve, Peru.

**FIGURE 2:**
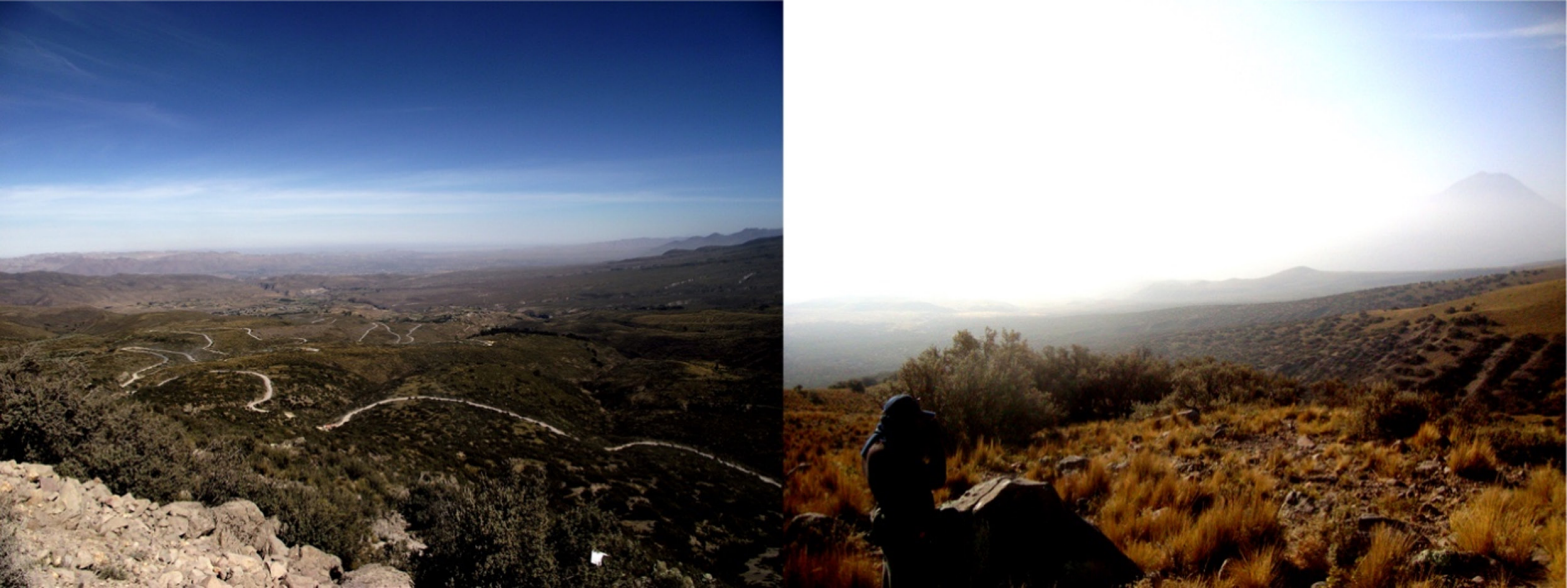
*Polylepis* forests in “El Simbral” (left) and “Tuctumpaya” (right), Arequipa, Peru.

Fieldwork was conducted within the *Polylepis* forests (*Polylepis rugulosa* Bitter 1911) in the buffer zone of the Salinas y Aguada Blanca National Reserve (SABNR), Arequipa, Peru (Figure 1). The habitat is comprised of shrub‐like vegetation, including *Adesmia spinossisima, Baccharis* spp., *Chuqiraga rotundifolia*, *Mutisia acuminata*, *Parasthrephia lepidophylla*, *Senecio graveolens*, and *S. nutans*, while herbaceous species include *Belloa piptolepis*, *Calamagrostis breviaristata*, *Festuca orthophylla*, *Gnaphalium purpureum*, *Sysirinchium chilense*, and *Werneria aretioides* (Mendoza et al., 2010). We studied the lizard populations of two distinct areas, locally called “El Simbral” and “Tuctumpaya.” Both of the areas are located within the Pichu Pichu volcano's lowland area, which has been subject to various anthropogenic pressures. El Simbral is located in the Chiguata District in Arequipa. It is comprised of *Polylepis* forest fragmented by a road passing through the Moquegua and Puno Regions of Southern Peru (Figure 2). Tuctumpaya is located in the Pocsi District of Arequipa. In contrast, the *Polylepis* forest here is continuous, with a higher average tree height compared to El Simbral (Figure 2). The two areas provide an ideal comparison for studying the effects of fragmentation on the populations inhabiting them.

### 2.2 Methods

All data were collected between July and November 2013, within the dry season in the SABNR. We followed the Type I design for resource selection studies to collect data on resource use and resource availability at the population level (Manly et al., 2002). For both El Simbral and Tuctumpaya, we surveyed 30 quadrats of 20 × 20 m, within a total area of ~475 Ha and between an elevation range of 3600 and 4200 m. These quadrats were randomly selected using QGIS version 1.8.0. In each quadrat, we determined the abundance of nine microhabitats: (1) *Polylepis* trees, (2) scrubland, (3) thorny bushes, (4) nonthorny bushes, (5) rocks (major diameter > 1 m), (6) small rocks (major diameter ≤ 1 m), (7) uncovered land, (8) dry organic matter, and (9) *Nordenstamia longistyla*. *Nordenstamia longistyla* is an Asteraceae shrub which was abundant in our study area. We estimated the coverage of each microhabitat in the quadrats using the Crown‐Diameter method (Mueller‐Dombois & Ellenberg, 1974) and calculated areas using the ellipse equation (see García‐De la Peña et al., 2012). Uncovered land coverage was then calculated by subtracting the total area of the quadrat from the combined area of the microhabitats. In each area, the microhabitat coverages were averaged. The final means were considered to be estimates of the relative abundances of each resource within the forests and, therefore, a measure of their availability (Manly et al., 2002).

*Liolaemus etheridgei* individuals were searched for using the visual encounter survey (McDiarmid et al., 2012). Each quadrat was surveyed once, at a randomly assigned time between 8:00 and 17:00 h. In all quadrats, surveys were carried out by two observers who walked, one behind the other, in a zigzag pattern across the quadrat for 30 ± 5 min. We recorded the microhabitat of where each individual was encountered. We also recorded the time of encounter and the activity of the individual. In order to assess diet, several individuals were collected by hand, euthanized with Halatal, fixed in 10% formalin, and preserved in 70% ethanol. In addition, to study prey availability, we randomly installed six lines of pitfall traps (20 traps separated 10 m apart) in El Simbral and Tuctumpaya. The pitfall traps consisted of 1 L plastic containers filled with 0.5 L of water plus washing up liquid. Pitfall trap lines were left for 48 h; then, the specimens were collected and preserved in 70% ethanol. Specimens collected were deposited in the scientific collection of the Herpetology and Entomology Departments of the Museo de Historia Natural de la Universidad San Agustín de Arequipa (MUSA) in Peru.

### 2.3 Laboratory

To study the diet of *L. etheridgei*, 33 adult specimens were dissected (El Simbral: four males + 10 females = 14, Tuctumpaya: eight males + 11 females = 19), and their stomach contents extracted. Most of the stomach items were identified at family level using specialized bibliography (Borror et al., 1981) and by comparison to reference material collected in the pitfall traps. However, some were identified until a higher taxonomical level (see Table 2). Amongst the contents, plant material was also present. Thus, all taxonomical items plus the plant material were considered prey categories like in similar lizard diet studies (e.g. Semhan et al., 2013). Dry weight measurements were taken for each prey category using a digital scale (Anyload ES‐203HA precision 200 g × 0.001 g).

### 2.4 Data analyses

We assessed the microhabitat use with selection ratios: $W_{i}=u_{i}/a_{i}$, where $u_{i}$ and $a_{i}$ are the proportions of use and availability of resource i, respectively. We also determined the 95% confidence intervals for each $W_{i}$ value using a conservative Bonferroni adjustment (Manly et al., 2002). If the confidence intervals of $W_{i}$ were >1, the resource use was greater than its availability, so it was interpreted as a positive selection. If they were <1, there was a negative selection for the respective microhabitat, so the resource was avoided. When the confidence intervals of $W_{i}$ included 1, there was neither a positive nor negative selection; thus, the individual was indifferent to the microhabitat. In addition, we used the log‐likelihood of Pearson's statistic $\left(X_{L}^{2}\right)$ to statistically test for the existence of a selective process in El Simbral and Tucutmpaya as well as for each particular microhabitat; the latter included a Bonferroni adjustment (Manly et al., 2002). Lastly, to test the selection process for microhabitats, we also estimated Manly's standardized selection index ($B_{i}$), which gives the probability that a resource is used when others are equally available (Manly et al., 2002). The above analyses were done using the adehabitatHS R package version 0.3.14 (Calenge, 2006).

Daily activity pattern was represented with frequency bar charts and kernel density charts considering two contrasting behaviors: active and inactive. We also tested for differences in the abundances of individuals from El Simbral and Tuctumpaya using the Wilcoxon signed‐ranks test (Aho, 2014). This test was also used to compare the abundance of active and inactive individuals in El Simbral and Tuctumpaya throughout the day.

For each category of prey, we obtained the number of individuals (*N*), frequency of stomachs containing the prey (*F*), and weight (*W*). However, for plant material, we only estimated the *F* and *W*; furthermore, this item was not included in any further analysis. We determined the percentage of each measurement (%*N*, %*F*, %*W*). With these measurements, we obtained the Relative Importance Index (IRI; Pinkas, 1971) but used the weight instead of the prey volume (Martin et al., 1996). Thus, the index is IRI = (%*N* + %*W*)*%*F*. The IRI is used mainly in fishery studies but is also recommended in other taxa, such as reptiles (Hart et al., 2002). We used the hierarchized %IRI values according to the categories proposed by Montori (1991), which have been used in diet studies in other *Liolaemus* species (see Semhan & Halloy, 2016). Thus, prey categories were ordered under the following dietary hierarchy: fundamental (*F*: %IRI > 75%), secondary (S: 75% > %IRI > 50%), accessory (A: 50% > %IRI > 25%), and accidental (a: 25% > %IRI). This hierarchy was originally proposed for values of the “Lambda segunda” dominance index (Montori, 1991); however, it has regularly been used with other indexes in dietary studies of *Liolaemus* species (see Cabrera & Scrocchi Manfrini, 2020; Olivera Jara & Aguilar, 2020; Semhan & Halloy, 2016). Trophic niche amplitude was estimated using the standardized Levin's index: $B_{a}=\left(1/\left(\sum_{i=1}^{n}p_{i}^{2}\right)-1\right)/\left(n-1\right)$, where $p_{i}$ is the proportion of prey category *i*, and n is the total number of prey categories (Krebs, 1999). We obtained the proportions under the “averaged” method as it reduced the bias of individuals that eat a large number of items (Zaccarelli et al., 2013). We excluded plant material in all the above analyses as it was not convenient to estimate *N* and %*N*. However, we re‐estimated the %*W* to consider the plant content and then determined the type of diet according to the %*W* of plant material using the scale of Espinoza et al. (2004): insectivorous (0–10%), omnivorous (11–50%), and herbivorous (51–100%). Using data obtained from the pitfall traps, we determined the effective number of species (Jost, 2006) for both areas and the relative abundance of each prey category; the latter was considered a measure of prey availability in the forests. Then, we estimated the selection ratios considering each prey category as a food resource and the %*N* as a measure of use. In addition, we determined the standardized Manly's selection ratio with a type I design (Manly et al., 2002). To test for statistical differences in the diet of the two populations, we applied a permutational multivariate analysis of variance (Permanova) using a Bray–Curtis dissimilarity (Anderson, 2001) and a square root data transformation. As a Permanova assumes similar multivariate distribution (Anderson, 2001), we tested the multivariate homogeneity of group dispersions using the “betadisper” function from the “vegan” R package version 2.5‐5 (Oksanen et al., 2013). Finally, we used non‐metric multidimensional scaling (Zuur et al., 2007) to order and represent the prey categories consumed by *L. etheridgei* in each *Polylepis* forest. We complemented the above analysis with a Pianka overlap index: $O_{j,k}=\sum_{i}^{n}p_{i,j}p_{i,k}/\sqrt{\sum_{i}^{n}p_{i,j}^{2}\sum_{i}^{n}p_{i,k}^{2}}$, where $p_{i,j}$ is the proportion of prey category *i* of the total of preys eaten by species *j*, $p_{i,k}$ is the proportion of prey category *i* of the total of prey eaten by species *k*, and n is the total number of prey categories (Krebs, 1999). All data analyses were done in R version 3.5.2 (R Core Team, 2019).

## 3 RESULTS

### 3.1 Microhabitat use

**FIGURE 3:**
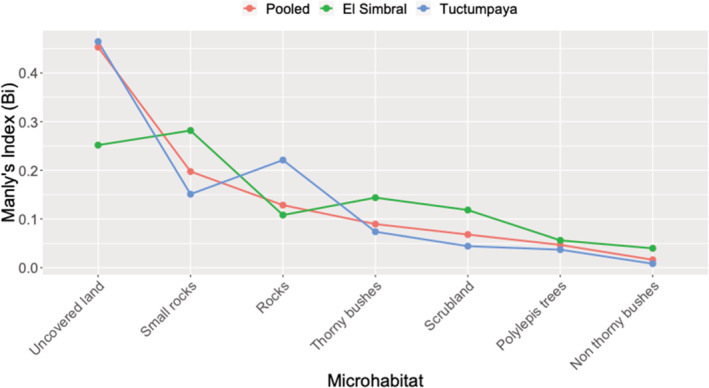
Manly's index for microhabitats used by *Liolaemus etheridgei* in *Polylepys* forest of El Simbral and Tuctumpaya, Arequipa, Peru.

We recorded 168 individuals of *L. etheridgei* across both areas and amongst multiple microhabitats (El Simbral: 59, Tuctumpaya: 109). The microhabitats *Nordenstamia longistyla* and dry organic matter were not used by *L. etheridgei*, so they were excluded from further analysis. We found that *L. etheridgei* exhibited selection for microhabitats in general ($X_{L}^{2}$ = 29.466, *p* < .01). Positive selection was not found for any microhabitats, whereas negative selection (i.e., avoidance) was found for *Polylepis* trees and nonthorny bushes. We did not find evidence of selection for uncovered land, small rocks, rocks, thorny bushes, or scrubland (Table 1). The Manly's index indicated that, amongst all microhabitats assessed, uncovered land would be the most attractive ($B_{i}=0.453$) and nonthorny bushes would be the least attractive ($B_{i}=0.016$) for *L. etheridgei* (Figure 3).

**TABLE 1:** Microhabitat selection of *Liolaemus etheridgei* in El Simbral and Tuctumpaya *Polylepis* forests, Arequipa, Peru.

Microhabitat	Pooled						El Simbral						Tuctumpaya					
	Available	Used	*W*_*i*_	*SE*	95% CI	*p*‐Value	Available	Used	*W*_*i*_	*SE*	95% CI	*p*‐Value	Available	Used	*W*_*i*_	*SE*	95% CI	*p*‐Value
*Polylepis* trees	0.351	0.149	0.424	0.125	0.118–0.73	<.007	0.293	0.119	0.405	0.157	0.02–0.79	<.007	0.409	0.165	0.404	0.100	0.16–0.648	<.007
Scrubland	0.155	0.095	0.613	0.251	0.003–1229	.124	0.159	0.136	0.855	0.344	0.012–1.698	.673	0.152	0.073	0.483	0.200	0.000–0.973	.010
Thorny bushes	0.096	0.077	0.807	0.393	0.00–1.77	.624	0.147	0.153	1.038	0.407	0.042–2.034	.925	0.046	0.037	0.804	0.539	0.000–2.125	.716
Non thorny bushes	0.081	0.012	0.147	0.154	0.00–0.525	<.007	0.059	0.017	0.288	0.308	0.000–1.043	.021	0.103	0.009	0.089	0.093	0.0663–0.1117	<.007
Rocks	0.057	0.065	1.159	0.674	0.000–2.811	.814	0.087	0.068	0.780	0.455	0.000–1.894	.629	0.027	0.064	2.411	1.704	0.000–6.586	.408
Small rocks	0.200	0.357	1.783	0.443	0.698–2.868	.077	0.200	0.407	2.034	0.520	0.759–3.309	.047	0.201	0.330	1.646	0.398	0.671–2.621	.105
Uncovered land	0.060	0.244	4.089	1.810	0.000–8.523	.088	0.056	0.102	1.818	1.031	0.000–4.345	.428	0.063	0.321	5.066	2.072	0.000–10.143	.050

*Note*: Available and used data are expressed as proportions. In all cases, *p*‐values are compared with Bonferroni level = 0.007.

In El Simbral, there was no positive selection (*W*_*i*_ ± CI 95% > 1) for any of the microhabitats; however, there was negative selection for *Polylepis* trees (*W*_*i*_ ± CI 95% < 1). We did not detect any selection processes for nonthorny bushes, rocks, scrubland, small rocks, thorny bushes, or uncovered land (Table 1). In Tuctumpaya, we did not detect a positive selection, yet there was negative selection for *Polylepis* trees and nonthorny bushes (*W*_*i*_ ± CI 95% < 1). There was no selection for rocks, scrubland, small rocks, thorny bushes, or uncovered land (Table 1). Manly's standardized selection indices for small rocks, thorny bushes, *Polylepis* trees, and nonthorny bushes were higher in El Simbral, whereas the Tuctumpaya population showed a higher selection probability for uncovered land and rocks (Figure 3).

### 3.2 Daily activity pattern

**FIGURE 4:**
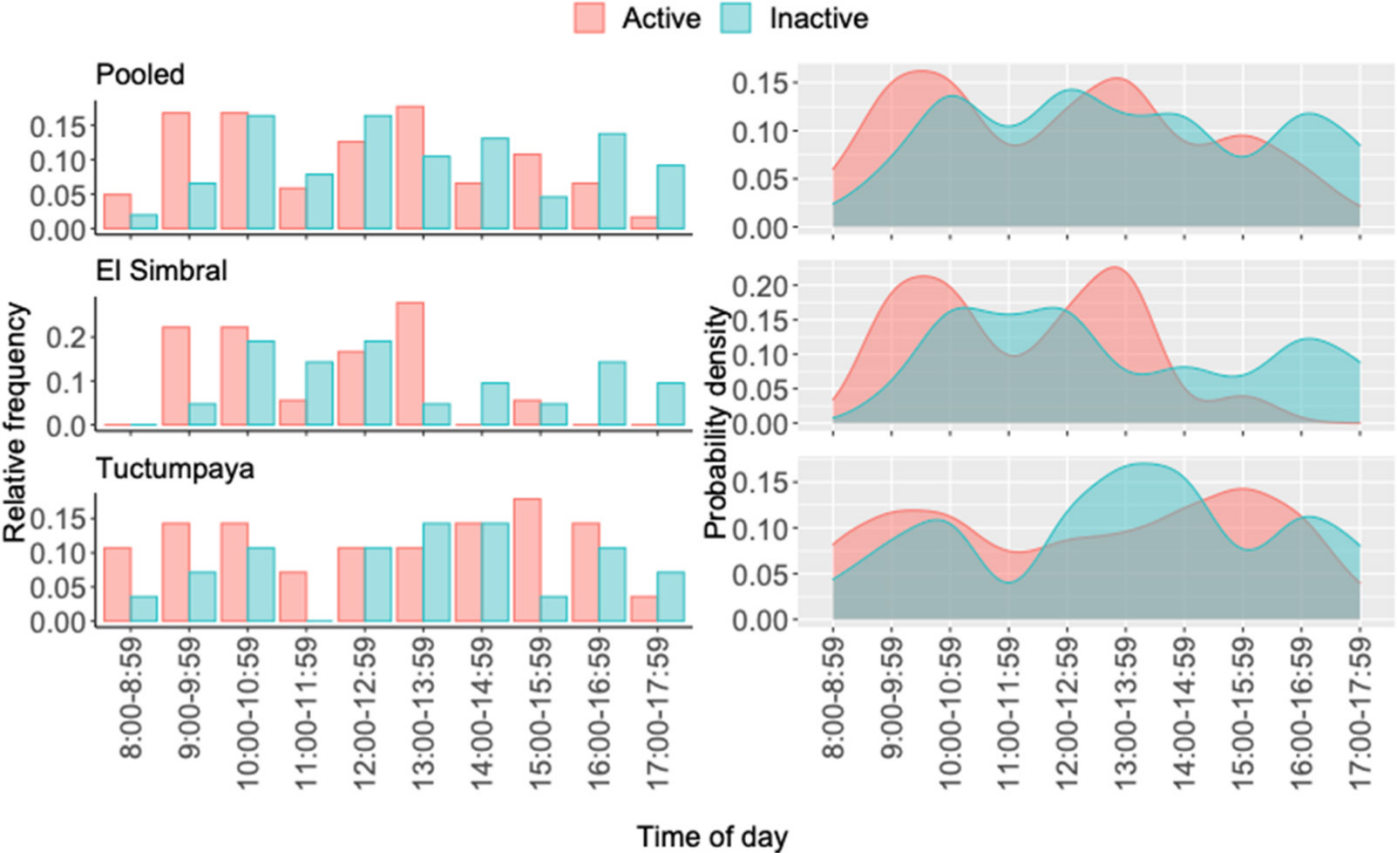
Daily activity pattern of *L. etheridgei* in the *Polylepis* forests of El Simbral and Tuctumpaya, Arequipa, Peru. Probability density functions are based on univariate kernel estimator (bandwidth = 1/3).

We found that *L. etheridgei* had a bimodal daily activity pattern, with one activity spike between 9:00 and 10:59 h and another between 13:00 and 13:59 h (Figure 4). *Liolaemus etheridgei* was also active at different times between sites, showing activity between 9:00 and 15:59 h in El Simbral and between 8:00 and 17:59 h in Tuctumpaya (Figure 4). The probability density functions revealed a tendency for a bimodal activity pattern in El Simbral, with one spike between 9:00 and 10:59 h and another between 13:00 and 13:59 h. The bimodal pattern was less evident in Tuctumpaya, where there was one activity peak between 9:00 and 10:59 h and another between 15:00 and 15:59 h. In both cases, there was a drop in activity between 11:00 and 11:59 h (Figure 4).

We did not find differences in the abundances of active and inactive individuals at different hours of the day for either the El Simbral (Wilcoxon signed‐rank test, *N =* 10, *V =* 21, *p =* .906) or Tuctumpaya populations (Wilcoxon signed‐rank test, *N =* 10, *V =* 30, *p =* .105). Populations from El Simbral and Tuctumpaya did not differ in their abundances of active (Wilcoxon signed‐rank test, *N =* 20, *V =* 22, *p =* .61) or inactive (Wilcoxon signed‐rank test, *N =* 20, *V =* 33, *p =* .61) individuals throughout the day.

### 3.3 Diet

The trophic niche breadth of *L. etheridgei* was 0.128. According to the IRI index, we found that Lygaeidae is the most important animalian dietary item for *L. etheridgei* in the *Polylepis* forests, being classified as a “Secondary” prey (% IRI: 75% > 55.3% > 50%). All other items fell into the category of “Accidental” (%IRI < 25%). Due to the weight of plant material, *L. etheridgei* was found to be herbivorous (%*W* Plant material: 66.373, >51%). In addition, prey selection was found ($X_{L}^{2}$ = 944.486, *p* < .05). The selection ratios indicated that *L. etheridgei* selects positively for Lygaeidae (*W*_*i*_ = 14.675 ± 4.456, *p* < .002), whereas it selects negatively against Anthomyiidae (*W*_*i*_ = 0.166 ± 0.111, *p* < .002), Formicidae (*W*_*i*_ = 0.028 ± 0.056, *p* < .002), Cicadelidae (*W*_*i*_ = 0.142 ± 0.199, *p* < .002), Drosophilidae (*W*_*i*_ = 0.028 ± 0.078, *p* < .002), and Tephritidae (*W*_*i*_ = 0.231 ± 0.655, *p* < .002), (*p* values compared with Bonferroni level = 0.002) (Figure 6). The confidence intervals of the remaining prey categories included the critical value 1; thus, there was no selective process for those food items.

**FIGURE 5:**
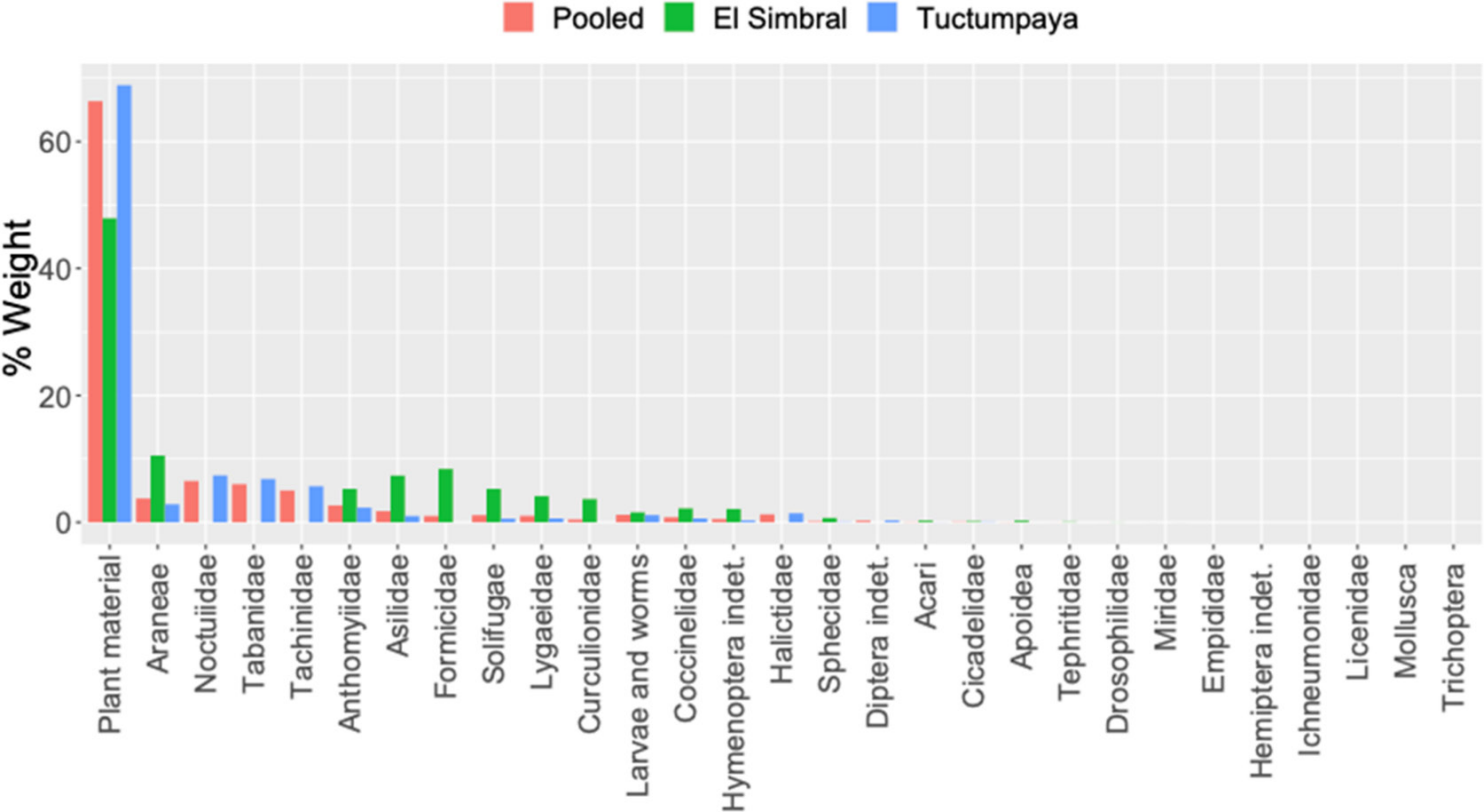
Dry weight percentage contribution of prey categories to the diet of *Liolaemus etheridgei* in *Polylepis* forest of El Simbral and Tuctumpaya, Arequipa, Peru. Percentage of plant material: insectivorous (0–10%), omnivorous (11–50%), herbivorous (51–100%).

**FIGURE 6:**
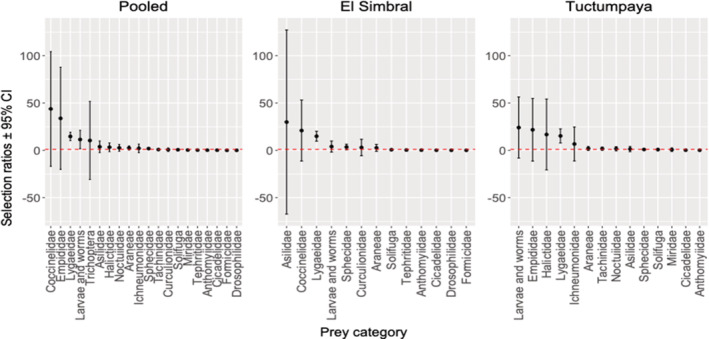
Resource selection ratios for preys consumed by *L. etheridgei* in *Polylepis* forest of El Simbral and Tuctumpaya, Arequipa, Peru. The dashed red line indicates the critic value 1.

Trophic niche breadth was broader in Tuctumpaya ($B_{a}$ = 0.202) than in El Simbral ($B_{a}$ = 0.147). Lygaeidae (Hemiptera) was the most important prey for both sites, with this prey being “Secondary” in El Simbral (%IRI: 62.9%) and “Accesorial” in Tuctumpaya (%IRI: 44.8%). The remaining items were considered as “Accidental” (%IRI: <25%) (Table 2). The plant material weights indicated that *L. etheridgei* is omnivorous in El Simbral (%*W* Plant material: 47.924%, between 11% and 50%) and herbivorous in Tuctumpaya (%*W* Plant material: 68.865%, >51%). In both cases, no other prey category surpassed 15% (Figure 5). The effective number of species from the pitfall traps was higher in El Simbral (14.52, *q* = 1) than in Tuctumpaya (11.38, *q* = 1). The selection ratios show that the El Simbral population selected positively for Lygaeidae (*W*_*i*_ = 14.941 ± 5.245, *p* < .004), whereas there was negative selection against Anthomyiidae (*W*_*i*_ = 0.215 ± 0.283, *p* < .004), Formicidae (*W*_*i*_ = 0.041 ± 0.076, *p* < .004), Cicadelidae (*W*_*i*_ = 0.09 ± 0.239, *p* < .004), and Drosophilidae (*W*_*i*_ = 0.057 ± 0.151, *p* < .004), (*p* values compared with Bonferroni level = 0.004). In the Tuctumpaya population, we found positive selection for Lygaeidae (*W*_*i*_ = 15.224 ± 7.481, *p* < .004), whereas there was negative selection against Anthomyiidae (*W*_*i*_ = 0.11 ± 0.08, *p* < .004) and Cicadelidae (*W*_*i*_ = 0.157 ± 0.244, *p* < .004; *p* values compared with Bonferroni level = 0.004). There was no selection for the unmentioned prey categories in either study site (Figure 6).

**TABLE 2:** Diet composition of *Liolaemus etheridgei* in El Simbral and Tuctumpaya *Polylepis* forests, Arequipa, Peru.

Prey category	El Simbral (*N* = 14)								Tuctumpaya (*N* = 19)								Pooled (*N* = 33)							
	*N*	%*N*	*F*	%*F*	*W*	%*W*	IRI	%IRI	*N*	%*N*	*F*	%*F*	*W*	%*W*	IRI	%IRI	*N*	%*N*	*F*	%*F*	*W*	%*W*	IRI	%IRI
Lygaeidae	76	46.9	13	92.9	0.0079	8.0	5094.6	62.9	82	35.5	17	89.5	0.0085	1.9	3349.5	44.8	158	40.2	30	20.7	0.016	3.0	894.8	55.3
Acari^a^	38	23.5	4	28.6	0.0005	0.5	684.8	8.5	21	9.1	10	52.6	0.0003	0.1	481.8	6.4	59	15.0	14	9.7	0.001	0.1	146.4	9.1
Araneae^a^	4	2.5	4	28.6	0.0200	20.2	648.4	8.0	8	3.5	6	31.6	0.0400	9.1	398.0	5.3	12	3.1	10	6.9	0.060	11.2	98.2	6.1
Sphecidae	11	6.8	6	42.9	0.0013	1.3	347.1	4.3	8	3.5	6	31.6	0.0009	0.2	116.2	1.6	19	4.8	12	8.3	0.002	0.4	43.5	2.7
Solifugae^a^	5	3.1	3	21.4	0.0100	10.1	282.8	3.5	4	1.7	2	10.5	0.0080	1.8	37.5	0.5	9	2.3	5	3.4	0.018	3.4	19.5	1.2
Anthomyiidae	4	2.5	3	21.4	0.0100	10.1	269.6	3.3	13	5.6	7	36.8	0.0325	7.4	480.9	6.4	17	4.3	10	6.9	0.043	7.9	84.5	5.2
Coccinelidae	7	4.3	3	21.4	0.0042	4.2	183.6	2.3	14	6.1	7	36.8	0.0084	1.9	294.0	3.9	21	5.3	10	6.9	0.013	2.3	53.0	3.3
Asilidae	2	1.2	2	14.3	0.0140	14.2	219.9	2.7	2	0.9	2	10.5	0.0140	3.2	42.8	0.6	4	1.0	4	2.8	0.028	5.2	17.2	1.1
Larvae and worms^a^	4	2.5	3	21.4	0.0030	3.0	117.9	1.5	18	7.8	7	36.8	0.0160	3.7	421.8	5.6	22	5.6	10	6.9	0.019	3.5	63.0	3.9
Formicidae	2	1.2	1	7.1	0.0160	16.2	124.4	1.5	–	0.0	–	–	–	–	–	–	2	0.5	1	0.7	0.016	3.0	2.4	0.1
Curculionidae	1	0.6	1	7.1	0.0070	7.1	55.0	0.7	–	0.0	–	–	–	–	–	–	1	0.3	1	0.7	0.007	1.3	1.1	0.1
Hymenoptera indet.^a^	3	1.9	1	7.1	0.0040	4.0	42.1	0.5	1	0.4	1	5.3	0.0040	0.9	7.1	0.1	4	1.0	2	1.4	0.008	1.5	3.5	0.2
Apoidea^a^	1	0.6	1	7.1	0.0005	0.5	8.0	0.1	–	0.0	–	–	–	–	–	–	1	0.3	1	0.7	0.001	0.1	0.2	0.0
Cicadelidae	1	0.6	1	7.1	0.0003	0.3	6.6	0.1	3	1.3	3	15.8	0.0009	0.2	23.8	0.3	4	1.0	4	2.8	0.001	0.2	3.4	0.2
Tephritidae	1	0.6	1	7.1	0.0002	0.2	5.5	0.1	–	0.0	–	–	–	–	–	–	1	0.3	1	0.7	0.000	0.0	0.2	0.0
Drosophilidae	1	0.6	1	7.1	0.0001	0.1	4.9	0.1	–	0.0	–	–	–	–	–	–	1	0.3	1	0.7	0.000	0.0	0.2	0.0
Diptera indet.^a^	1	0.6	1	7.1	0.0000	0.0	4.4	0.1	13	5.6	2	10.5	0.0040	0.9	68.9	0.9	14	3.6	3	2.1	0.004	0.7	8.9	0.6
Noctuiidae	–	–	–	–	–	–	–		5	2.2	4	21.1	0.1040	23.8	545.9	7.3	5	1.3	4	2.8	0.104	19.4	57.0	3.5
Tabanidae	–	–	–	–	–	–	–		4	1.7	4	21.1	0.0960	21.9	498.3	6.7	4	1.0	4	2.8	0.096	17.9	52.2	3.2
Tachinidae	–	–	–	–	–	–	–		10	4.3	4	21.1	0.0800	18.3	476.0	6.4	10	2.5	4	2.8	0.080	14.9	48.2	3.0
Empididae	–	–	–	–	–	–	–		13	5.6	5	26.3	0.0000	0.0	148.1	2.0	13	3.3	5	3.4	0.000	0.0	11.4	0.7
Halictidae	–	–	–	–	–	–	–		5	2.2	2	10.5	0.0200	4.6	70.9	0.9	5	1.3	2	1.4	0.020	3.7	6.9	0.4
Ichneumonidae	–	–	–	–	–	–	–		2	0.9	2	10.5	0.0000	0.0	9.1	0.1	2	0.5	2	1.4	0.000	0.0	0.7	0.0
Miridae	–	–	–	–	–	–	–		1	0.4	1	5.3	0.0001	0.0	2.4	0.0	1	0.3	1	0.7	0.000	0.0	0.2	0.0
Hemiptera indet.^a^	–	–	–	–	–	–	–		1	0.4	1	5.3	0.0000	0.0	2.3	0.0	1	0.3	1	0.7	0.000	0.0	0.2	0.0
Licenidae	–	–	–	–	–	–	–		1	0.4	1	5.3	0.0000	0.0	2.3	0.0	1	0.3	1	0.7	0.000	0.0	0.2	0.0
Mollusca^a^	–	–	–	–	–	–	–		1	0.4	1	5.3	0.0000	0.0	2.3	0.0	1	0.3	1	0.7	0.000	0.0	0.2	0.0
Trichoptera^a^	–	–	–	–	–	–	–		1	0.4	1	5.3	0.0000	0.0	2.3	0.0	1	0.3	1	0.7	0.000	0.0	0.2	0.0

Abbreviations: %*F*, Item frequency percentage; %IRI, Standardized index of relative importance; %*N*, Item number percentage; %*W*, Weight percentage; *F*, Item frequency; IRI, Index of relative importance; *N*, Item number; *W*, Weight.

^a^ Prey categories in higher taxonomical level than family.

**FIGURE 7:**
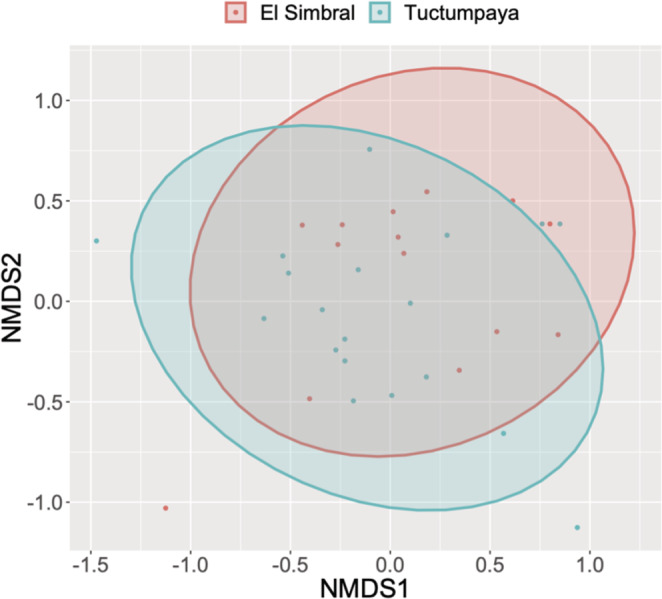
Unconstrained ordination of diet data based on non‐metric multidimensional scaling (stress = 0.194).

As multivariate homogeneity was found in the group dispersions (*F* = 0.411, *p* = .529, permutations = 9999), the Permanova analysis was used. The Permanova analysis showed no significant differences in diet between the El Simbral and Tuctumpaya populations (*F* = 1.036, *p* = .409, permutations = 9999), which is consistent with the high overlap value of the Pianka index ($O_{j,k}$ = 0.963) and the ordination obtained by the non‐metric multidimensional scaling (Figure 7).

## 4 DISCUSSION

### 4.1 Microhabitat use

Our results show that *L. etheridgei* does not select positively for any microhabitats found in the *Polylepis* forests; thus, this lizard can be considered a generalist in its habitat use (Vivas et al., 2019). The rocks, small rocks, and uncovered land have been associated with the thermoregulatory behavior of *L. quilmes*, *L. ramirezae* (Robles & Halloy, 2008), and *L. tenuis* (Victoriano et al., 2008). However, the absence of selective use for such resources may indicate that *L. etheridgei* does not use them actively for thermoregulation, which could support the idea that this lizard is a thermal generalist (Llanqui, 2020). The microhabitats' thorny bushes and nonthorny bushes could serve as refuges, shelters, or foraging sites. For instance, *L. multimaculatus* prefers tussocks of grass for shelters (Kacoliris et al., 2010), *L. quilmes* has been found in rocks close to shrubs (Robles & Halloy, 2008), and *L. lemniscatus* has been found in herbaceous patches (Jaksić et al., 1980). However, *L. etheridgei* was indifferent to scrubland, thorny bushes, and nonthorny bushes so that they could be used indistinctly as refuges, shelters, or foraging sites. This could be supported by our field observations of cavities used by *L. etheridgei*, which were encountered under different substrates such as rocks, thorny bushes, nonthorny bushes, and scrubland. Contrary to our expectations, *Polylepis* trees were avoided by *L. etheridgei* (negative selection). As there is no microhabitat positively selected by *L. etheridgei*, and this species may even avoid *Polylepis* trees, it is questionable as to why it inhabits these forests. Considering that *L. etheridgei* also inhabits sites where *Polylepis* trees are absent (Laurent, 1998), we suggest that *L. etheridgei* is inhabiting *Polylepis* forests perhaps simply because they occur within its elevational range. It could also imply that *L. etheridgei* is able to tolerate variation in habitat structure, which is consistent with our conclusion that species is a generalist. Moreover, this could explain there being almost no differences in microhabitat selection in El Simbral and Tuctumpaya as well as the apparent tolerance to fragmentation of the *Polylepis* forests.

### 4.2 Daily activity pattern

The activity peak of *L etheridgei*: from 9:00 to 10:59 h, is identical to that recorded for *L. qalaywa* (Chaparro et al., 2020), a species found near the distribution of *L. etheridgei*. Therefore, it is likely that both species would have a similar degree of solar radiation exposure and, accordingly, show a similar behavior pattern. The second activity peak occurs after midday: 13:00–13:59 h and is similar to that of *L. evaristoi*, a sister species of *L. etheridgei* (Gutiérrez et al., 2018). The drop in activity between 11:00 and 11:59 h supports a bimodal pattern for *L. etheridgei*. Bimodal activity patterns have also been recorded in *L. darwinii* (Videla & Puig, 1994), *L. koslowski* (Belver et al., 2010), *L. multimaculatus* (Vega et al., 2000), and *L. occipitalis* (Bujes & Verrastro, 2006, 2008). In particular, a bimodal activity with a drop around midday, like in *L. etheridgei*, has been recorded for *L. arambaresis* (Liz et al., 2019), *L. constanzae*, and *L. nigroroseus* (Labra et al., 2001), and this is thought to be a strategy in avoiding overheating. Throughout the day, there was no significant difference between the number of active and inactive individuals. Thus, there were no particular hours where active individuals were more abundant than inactive or vice versa, which is probably not valid in the late afternoon when the number of inactive lizards is logically greater than active ones. However, the actual number of inactive individuals could have been underestimated by the fact that the lizards would tend to seek shelter during periods of inactivity and thus would be harder to detect. This would likely have been near‐surface shelter between periods of high activity; however, deeper refuges may have been sought later in the day, further reducing detectability. The lack of differences between active and inactive individuals would suggest that the variation between the *Polylepis* forests, particularly the abundance cover, appears not to affect the activity patterns of *L. etheridgei* significantly. The range of daily activity of *L. etheridgei* was shorter in El Simbral than in Tuctumpaya, where the former began activity later (9:00 h) than in Tuctumpaya (8:00 h), and ended earlier (16:00 vs. 17:59 h). This result contradicts our expectation that the Tuctumpaya population would have a shorter daily activity range due to a higher cover of trees than in El Simbral, limiting access to solar radiation. *Polylepis* trees occupy rough terrains with areas where the sunlight reaches late in the day, while windy conditions can affect the body temperature of lizards (see Bujes & Verrastro, 2008); thus, both could be involved in the variability of the starting hour of activity.

### 4.3 Diet

Lygaeidae: Hemiptera was found to be the most important dietary component for *L. etheridgei*, and this is the only item that was selected for positively in both *Polylepis* forests. Even though Hemiptera does not seem to be a typical choice in the diet of *Liolaemus* (Halloy et al., 2006), it has been recorded as an important item in the diet of *L. eleodori* (Astudillo et al., 2015), *L. quilmes*, *L. ramirezae* (Halloy et al., 2006; Robles & Halloy, 2008), and *L. ruibali* (Villavicencio et al., 2005). Formicidae has been recorded as an important component in the diet of several *Liolaemus* species (Halloy et al., 2006) such as *L. koslowskyi* (Aun & Martori, 1998); *L. wiegmannii* (Martori et al., 2002); *L. elongatus* (Quatrini et al., 2001); *L. pseudoanomalus* (Kozykariski et al., 2011); *L. cuyanus* (Moreno Azócar & Acosta, 2011); *L. irregularis*, *L. albiceps*, *L. multicolor*, and *L. yanalcu*, (Valdecantos, 2011). However, Formicidae was avoided by *L. etheridgei* and seems not to be an essential prey for this lizard. Interestingly, Mella et al. (2010) reported the avoidance of Formicidae and a positive selection for Lygaeidae in *L. puna* (= *L. barbarae*), although plant material was not found in the diet as it was in *L. etheridgei*. It is worth noting that *L. etheridgei* belongs to the *Eulaemus* subgenus while *L. puna* (=*barbarae*) belongs to the *Liolaemus* subgenus (Lobo et al., 2010), so this could be a case of ecological convergence. The avoidance of Cicadelidae, Drosophilidae and Tephritidae could be due to their high mobility which makes predation difficult despite their abundance, but more comprehensive studies are needed to assess this.

Based on the percentage of plant material found, *L. etheridgei* can be classified as herbivorous, which contradicts the classification of this lizard as mainly arthropophagous by Olivera‐Jara and Aguilar (2020). *L. etheridgei* would therefore belong to the 9.5% of species within the *Eulaemus* subgenus, considered herbivores (Cabrera & Scrocchi Manfrini, 2020). Analysis of diet within each forest found *L. etheridgei* to be herbivorous in Tuctumpaya and omnivorous in El Simbral, although the latter showed a percentage of plant consumption close to the critical value that separates omnivorous from herbivorous (Figure 5). Intraspecific variation in plant consumption has also been recorded for *L. elongatus* (Quatrini et al., 2001). Although we recommend additional surveys to confirm the diet of *L. etheridgei*, we think that herbivory or an intermediate consumption of plant material is evident. There are several *Liolaemus* species with an intermediate consumption of plants: *L. alticolor*, *L. jamesi*, and *L. signifier* (Valencia et al., 1982; cited in Semhan et al., 2013), *L. eleodori* (Astudillo et al., 2015), *L. pictus* (Vidal & Sabat, 2010), *L. polystictus* (Olivera Jara & Aguilar, 2020), amongst others (see Abdala et al., 2012). Semhan et al. (2013) point out the existence of a continuum between carnivory–omnivory–herbivory; accordingly, we suggest that *L. etheridgei* is not in any extreme but biased toward herbivory. Regarding the tendency for herbivory, *L. etheridgei* would be a “widely foraging” species (Huey & Pianka, 1981) as it would need to move in order to reach plants. We also noticed a sit‐and‐wait foraging strategy, which is suitable for catching highly mobile preys (e.g., Diptera), and this aligns with the foraging behaviors observed by Pearson (1954) in *Liolaemus* populations from Arequipa, Peru. Therefore, we suggest that *L. etheridgei* is an intermediate forager with a tendency to sit‐and‐wait, as has previously been proposed for *L. wiegmanni* (Aun et al., 1999). Plant consumption could be associated with Lygaeidae as they are Hemiptera which extract sap from plants. Thus, we outline two scenarios: (1) plant products are essential for *L. etheridgei*, and it has to consume insects associated with them, and therefore this species is mainly herbivorous or (2) *L. etheridgei* mainly consumes arthropods (e.g., Lygaeidae) but consumes vegetation accidentally while catching them. A more complex scenario would be that both plant material and Lygaeidae are consumed because they are of similar importance. As expected, we found the trophic niche breadth in Tuctumpaya to be higher than in El Simbral. However, the Permanova analysis showed no animalian dietary differences between the El Simbral and Tuctumpaya populations; moreover, Pianka's overlap index indicated a high overlap in diet. This led us to believe that the current fragmentation of these forests does not affect the diet of *L. etheridgei* significantly.

The three ecological aspects evaluated in this study are not independent, so it is important to interpret them jointly. The absence of selection for microhabitats in *Polylepis* forests suggests that *L. etheridgei* is a generalist and does not choose specific sites for heating or foraging. In lizards, microhabitat use is affected by environmental temperature, which varies across the day (Carothers et al., 1998). Thus, the frequent use of uncovered land in the morning (Appendix 1) seems to relate to the heliothermic strategy of *L. etheridgei* (Llanqui, 2020), which would gain heat by direct exposure to the sun until it achieves adequate temperatures to be active. Likewise, the bimodal pattern opens the question of whether microhabitats are used in the same way in the morning and afternoon activity peaks. However, more data and design type III (Manly et al., 2002) are necessary to test this hypothesis. *L. etheridgei* reaches its maximum body temperature (~30 °C) between 12:00 and 13:00 h (Llanqui, 2020), coinciding with the increase in activity in the afternoon. We think *L. etheridgei* is likely physiologically operative during these hours. The absence of microhabitat selection and the selective consumption of Lygaeidae: Hemiptera can be explained considering that this bug is not restricted to specific plants but rather dispersed in vegetation. Thus, *L. etheridgei* would not need to select foraging sites, and the prey selection process would occur at a fine spatial scale.

### 4.4 Final remarks

Given the complexity of studying the multiple ecological aspects of *L etheridgei*, we outline some variables that could affect our results. Firstly, several *Liolaemus* species shift their ecology in response to seasonality, including microhabitat use (Simonetti, 1984), daily activity patterns (Belver et al., 2010; Bujes & Verrastro, 2006, 2008; Cabrera & Scrocchi, 2014; Liz et al., 2019; Vega et al., 2000; Videla & Puig, 1994), and diet composition (Pincheira‐Donoso, 2012). Therefore, we believe that variations in the ecology of the *L. etheridgei* inhabiting *Polylepis* forests would likely occur through different seasons as the weather conditions change dramatically from summer to winter. Secondly, it is also plausible that juvenile individuals show different patterns in microhabitat selection, daily activity patterns (Cabrera & Scrocchi, 2014; Carothers et al., 1998), and diet (see Duarte Rocha, 1998; Rocha, 1999). Finally, reproductive state (see Llanqui, 2017) and sex have also been recognized to potentially lead to differences in activity patterns (Cabrera & Scrocchi, 2014; Duarte Rocha, 1998) and diet (Semhan et al., 2013). We would encourage future studies to consider the above factors to improve our understanding of *L. etheridgei* and *Liolaemus* species in general.

We conclude that *L. etheridgei* is a generalist species with little selection for most of the resources we identified in *Polylepis* forests. The absence of significant differences in the use of microhabitats, daily activity patterns, and diet between the populations of the fragmented and non‐fragmented forest also supports a generalist character that is unaltered by the variation of the habitat resources. We predict this generalist tendency is also present in populations living outside *Polylepis* forests, such as grasslands, thickets, and even near cities. Finally, we point out that *L. etheridgei* in the *Polylepis* forests represents an opportunity to ensure the conservation of the species in southern Peru by protecting the *Polylepis* forests of the RNSAB and its buffer zone.

## AUTHOR CONTRIBUTIONS

**Irbin B. Llanqui:** Conceptualization (lead); formal analysis (lead); investigation (equal); methodology (equal); writing – original draft (equal); writing – review and editing (equal). **Bryn Edwards:** Investigation (equal); methodology (equal); writing – original draft (equal); writing – review and editing (equal). **Evaristo López Tejeda:** Investigation (equal); methodology (equal); supervision (lead); writing – original draft (equal); writing – review and editing (equal).

## CONFLICT OF INTEREST

The authors declare no conflict of interest.

## Data Availability

Raw data: Dryad, https://doi.org/10.5061/dryad.31zcrjdnj.

## APPENDIX 1

Microhabitats used across the day by *Liolaemus etheridgei* in the *Polylepis* forests of Arequipa, Peru.
